# Hybrid caffeic acid derivatives as monoamine oxidases inhibitors: synthesis, radical scavenging activity, molecular docking studies and in silico ADMET analysis

**DOI:** 10.1186/s13065-018-0481-7

**Published:** 2018-11-09

**Authors:** Priyanka Dhiman, Neelam Malik, Anurag Khatkar

**Affiliations:** Laboratory for Preservation Technology and Enzyme Inhibition Studies, Faculty of Pharmaceutical Sciences, M. D. University, Rohtak, Haryana 124001 India

**Keywords:** Monoamine oxidase, In silico design, Caffeic acid derivatives, DPPH and H_2_O_2_ activity

## Abstract

**Background:**

Monoamine oxidase has been implicated in numerous neurological disorders. Although synthetic monoamine oxidase inhibitors (MAOI) have emerged with many side effects, the aspiration of natural based MAOI has greatly increased. As they exhibit fewer side effects and food interaction along with improved neuropharmacological profile.

**Results:**

The in silico design of the caffeic acid derivatives led potent MAO inhibitors with remarkable antioxidant activity. The mechanistic insight of the compounds within the hMAO active site was achieved by molecular docking which led us to be more confident of the possible inhibition of MAO.

**Conclusions:**

The synthesized eugenol based ester of caffeic acid compound **7** exhibited MAO-A inhibition with IC_50_ values of 07.03 ± 0.022 µM with good selectivity (SI = 0.291) towards MAO-A. Conversely, two anilides compounds **2** and **1**, bearing chloro and nitro group at 2, 4 positions showed MAO-A inhibition with IC_50_ values of 08.51 ± 0.017 µM and 08.87 ± 0.005 µM, respectively. Only one compound **5** was found as a significant MAO-B inhibitor with the IC_50_ value of 10.80 ± 0.024 µM. Moreover, compounds **1**, **2**, **4** and **9** have profoundly appeared as potent antioxidants as evaluated in duel assay by scavenging DPPH and H_2_O_2_.

## Background

The monoamine oxidases (MAO; EC 1.4.3.4.) are flavin adenine dinucleotide (FAD) including enzymes that present in the outer mitochondrial membranes of astrocytes and radial glia, catecholaminergic neurons, serotoninergic neurons and in other cells [1]. MAO renders the oxidative deamination of monoamine neurotransmitters (R-NH_2_), exerts the corresponding aldehydes and the byproducts (H_2_O_2_ and ammonia) [2]. These metabolic products in particular, H_2_O_2_, are neurotoxic and activate the generation of reactive oxygen species (ROS) and stimulate neuronal apoptosis through mitochondrial damage. The intensity of MAO-B expression in neuronal tissue enhance fourfold with the age, consequentially high level of dopamine catabolism generates the excess of hydrogen peroxide, which exerts the pathology of neurodegenerative disorders such as Parkinson’s and Alzheimer’s diseases [3–6]. An increased level of MAO-A in the brain has been reported cause depression and anxiety. Because of their essential role in the metabolism of neurotransmitters, thus MAO enzymes are characterized as remarkable drug targets in the neuropsychological therapy and neurodegenerative diseases [7, 8].

Nature has been always a source of new lead compounds and plays a crucial role to treat several diseases by providing lead structures for the development of new synthetic drug molecules [9]. Since 3D structure information of the complex structure of MAO is available, so the molecular docking model might help to explore the structural requirements for the pharmacophore complex [10]. Among the natural sources of MAO inhibitors, the class of phenolic compounds has been extensively studied along with computer-aided approaches such as docking simulations, quantum mechanics and COMFA [11, 12]. Phenolic acids such as ferulic acid, gallic acid, protocatechuic acid, trans-cinnamic acid, and ellagic acid have been investigated on rat and mice brain mitochondrial MAO inhibition (Table 1).

**Table 1:** MAO inhibition showed by different phenolic acids

Sr. no	Natural phenolic acid	Structure of MAO inhibitors	MAO inhibition value (µM)	References
1.	Ferulic acid		7.55 ± 0.49 hMAO-A 24.00 ± 1.98 hMAO-B	[13]
2.	Gallic acid		9.49 ± 0.83 hMAO-A	[14]
3.	Protocatechuic acid		300 µM rMAO-A 2411 µM rMAO-B	[15]
4.	Trans-Cinnamic acid		6.47 ± 0.73 rMAO-A 1.21 ± 0.071 rMAO-B	[16]
5.	Ellagic acid		412.24 nM rMAO-B	[17]

**Fig. 1:**
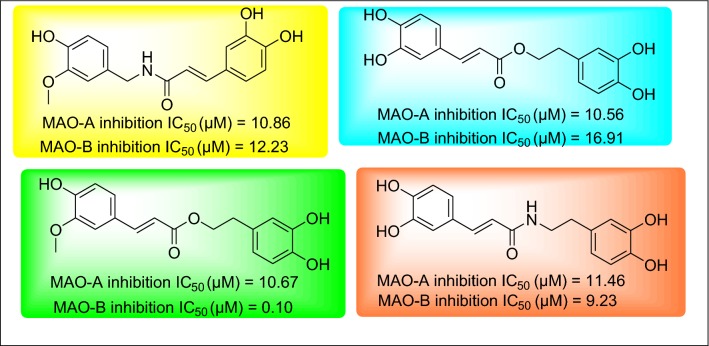
MAO inhibitory profile of caffeic acid derivatives found in the recent literature

The caffeic acid (3,4-dihydroxycinnamic) scaffold, which is abundant in nature, is tremendously resourceful and found as profoundly biological active molecules. Caffeic acid (CA) is one of the hydroxycinnamate and phenylpropanoid metabolites, widely distributed in plant tissues [18]. This polyphenol is present in many food sources, including coffee drinks, blueberries, apples, wine, and cider. CA and its derivatives including ethyl ester and phenyl ester (CAPE) are reported for various pharmacological activities, e.g. anticancer, antioxidant and neuroprotective properties [19]. There have been several studies aiming to investigate the neurological activates of caffeic acid derivatives. Takao and Coworkers evaluated the MAO inhibitory potential and free radical scavenging activity of amide and ester derivatives of caffeic acid (Fig. 1) [20].

Conversely, other study associated with decreased striatal dopamine levels due to loss of dopaminergic neurons in the substantia nigra. The caffeic acid phenethyl ester (CAPE) was observed as to be potent attenuator of neurodegeneration of dopaminergic neurons by inhibiting MAO-B at high concentrations 100 µM [21].

In a comparative study by Takeda et al. [22] evaluated the antidepressive-like effect of caffeic acid and rosmarinic acid by the forced swimming test in mice and evaluated the MAO inhibitory potential. Caffeic acid inhibited MAO-A activity up to 40.4%, with an IC_50_ > 1 mM. However, both of these phenolic acids did not produce significant monoamine oxidase-B inhibition [22].

More recently, Akomolafe and coworkers critically revealed the synergic potency of caffeine, caffeic acid on in vitro monoaminergic models for neurodegeneration in rat brain. Combination of caffeic acid with caffeine significantly reduced the level of MAO in the micromolar range [23]. Carpéné et al. 2015 published a computational docking study for caffeic acid by using Glide software. In the case of MAO-A, several aromatic – interactions appeared with Phe208, Tyr444, Tyr407, and Phe352. Maximum hydrogen bonds were formed between the hydroxyl groups of the ligand structures and polar amino acids Tyr444, Asn181 and Tyr197 [24].

**Fig. 2:**
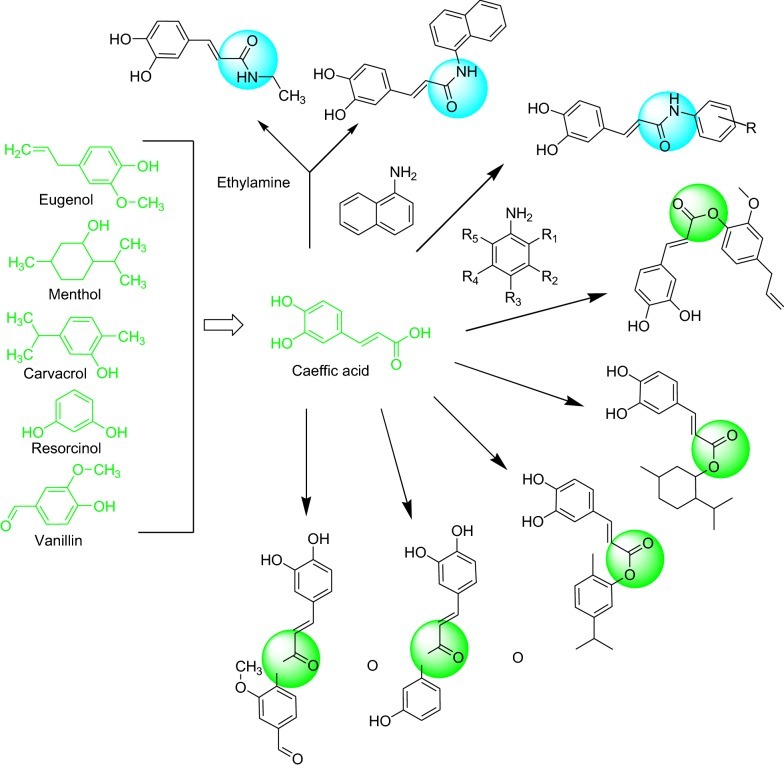
Design of strategy caffeic acid derivatives for anti-MAO and antioxidant activity

These evidence suggests that the caffeic acid as a useful candidate for the therapeutic management of neurological disorders. Thus, in the present study, we have synthesized and investigated the antioxidant and MAO inhibitory potential of novel caffeic acid derivatives with molecular docking (Fig. 2).

## Results and discussion

### Chemistry

**Scheme 1:**
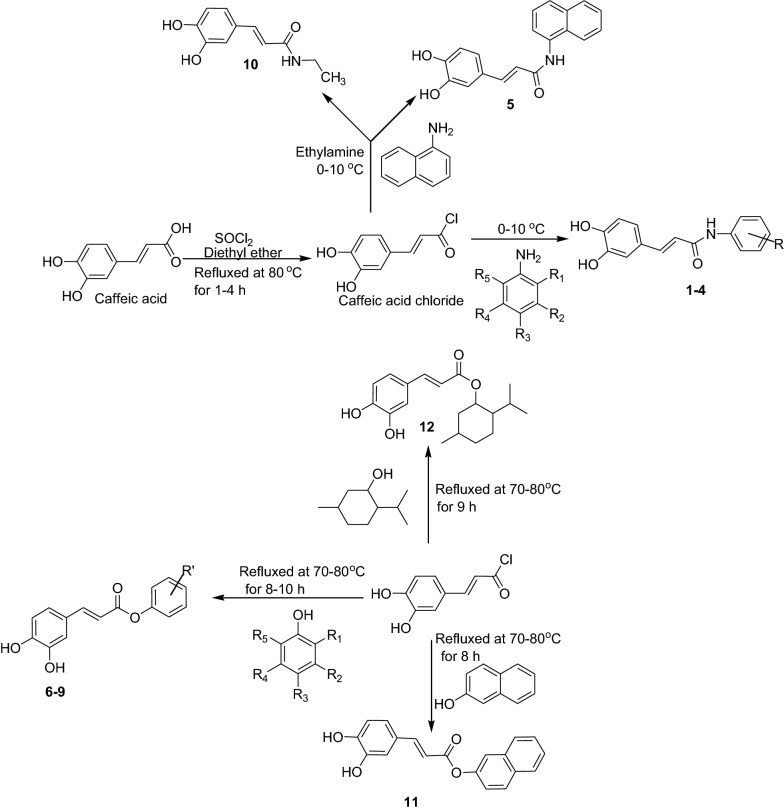
Synthetic route for the caffeic acid derivatives

A series of hybrid caffeic acid derivatives was synthesized according to reaction outlined in the Scheme 1. The chemical structures of all synthesized compounds were confirmed through IR, ^1^H NMR, ^13^C NMR, Mass spectroscopy and elemental analyses which were in full agreement with their structures. For the synthesis of intermediate caffeic acid chloride, the solution of caffeic acid and diethyl ether was refluxed with stirring at 80 °C for 1–4 h along with thionyl chloride in the presence of pyridine as the catalyst. Completion of reaction was detected by single spot TLC under UV lamp and IR: Formation of caffeic acid chloride was confirmed by peak shifted 1640 (carboxylic) to 1768 (acid chloride). The disappearance of 1H NMR singlet at 11.5 of caffeic acid also indicated the formation of acid chloride. Further, the natural hybrid caffeic acid esters were prepared by refluxing different natural aromatic and cyclic alcohols (Table 2). The evolution of HCl gas was stopped that confirmed the completion of reaction. The formation of esters was initially detected by IR that showed the shifting of acid peak to ester at around 1875 cm^−1^ for instance compound **8**. Moreover, the C^13^ NMR peak of esters generally appeared at 165–180 ppm for all of the ester compounds. The signals in the ^1^H NMR spectra of the particular protons of the hybrid compounds were interpreted by their coupling constants, chemical shifts and multiplicities. Disappearance of singlet at 5.0 of natural alcohol in the spectra during esterification reaction showed the formation of esters for instance compound **8**.

**Table 2:**
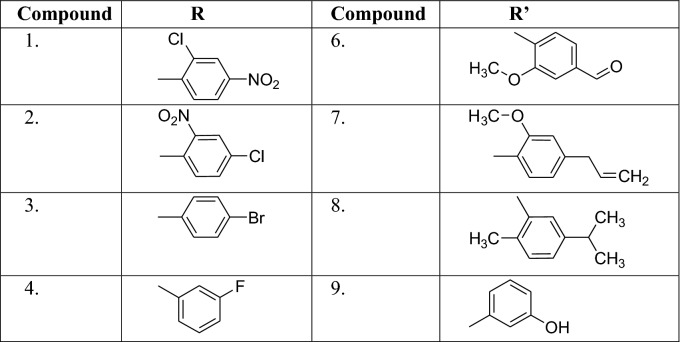
Substituent for the design of caffeic acid derivatives (1–12)

Preparation of amides was carried out by stirring of equivalent solutions of amine/aniline in ether dropped to a caffeic acid chloride solution in ether at 0–10 °C temperature up to 40 min. The resulted crude amide precipitates were acidified with 5% hydrochloric acid and then treated with 4% sodium carbonate to remove water and residual aniline finally the extracted anilides were recrystallized with methanol. The IR peak at 3354–3044 in amide compounds had shown the formation of N–H amide in **1**, **2**, **3**, **4**, **5** and **10**. Moreover, the stretching at 1627–1647 indicated the C=O amide formation in all amide compounds. In case of amides compound **1**, **2**, **3**, **4**, **5** and **10** the ^1^H NMR spectra showed amide proton peak at 8.67–7.60 ppm. However, the ^1^H NMR peak of aromatic C–NH_2_ at 4.2 was shifted to 8.61–7.40 indicated the formation of secondary amide NH in all amide compounds. In ^13^C spectra the amide derivatives showed phenolic carbons at 168.5–165.1 ppm. Finally, the mass spectroscopy was utilized for confirmation via determining molecular weight using Q-ToF Micro instrument as ion source. In positive chemical ionization most of the hybrid caffeic acid derivatives showed (M++1), M+ (molecular ion peak), (M++2) and in negative chemical ionization mode showed (M+1), (M+2), M+. The elemental analysis established the synthesis of hybrid caffeic acid derivatives where the % of C, H and N and in the synthesized compounds was observed to be within defined limits.

### MAO inhibitory activity

It has been recommended that the phenolic moiety in the parent structure is accountable for the MAO inhibitory action of the synthesized compounds. In relation to our investigational data, the majority of the compounds **(1**–**4)**, **(7**–**9)** and **12** inhibited MAO-A selectively. The mode of inhibition was established as competitive for all caffeic acid derivatives tested. According to the IC_50_ values experimentally found (Table 3), compounds **2** and **1**, which contains nitro and chloro substituent at the 2nd and 4th position, were found to be highly potent MAO-A inhibitors with IC_50_ values of 08.51 ± 0.017 µM and 08.87 ± 0.005 µM, respectively. MAO-A/MAO-B selectivity of compound **2** (bearing a 2-chloro, 4-nitro group) and **1** (bearing a 4-nitro, 2-chloro group) were found to be 0.191 and 0.209, respectively, shown their affinity towards MAO-A active site.

**Table 3:** Human MAO inhibitory activity of caffeic acid derivatives

Sr. no	IC_50_ (µM)^a^ hMAO-A	IC_50_ (µM)^a^ hMAO-B	Selectivity index^b^	Docking score hMAO-A	Docking score hMAO-B
1.	08.87 ± 0.005	42.34 ± 0.077	0.209	− 10.01	− 2.34
2.	08.51 ± 0.017	44.42 ± 0.014	0.191	− 11.11	− 3.45
3.	17.72 ± 0.006	21.14 ± 0.025	0.838	− 8.45	− 4.23
4.	18.47 ± 0.007	57.88 ± 0.029	0.319	− 7.12	− 3.87
5.	50.26 ± 0.035	10.80 ± 0.024	4.653	− 3.23	− 8.56
6.	20.36 ± 0.002	16.25 ± 0.035	1.252	− 6.56	− 7.53
7.	07.03 ± 0.022	24.14 ± 0.017	0.291	− 12.67	− 3.86
8.	19.75 ± 0.066	27.26 ± 0.072	0.724	− 6.12	− 5.34
9.	10.33 ± 0.012	27.25 ± 0.004	0.379	− 9.34	− 4.87
10.	25.22 ± 0.015	12.95 ± 0.046	1.947	− 4.72	− 8.45
11.	24.34 ± 0.011	21.48 ± 0.056	1.233	− 5.32	− 7.34
12.	22.02 ± 0.018	48.54 ± 0.077	0.453	− 6.27	− 3.86
Caffeic acid	11.72 ± 0.044	22.88 ± 0.009	0.512	− 10.63	− 7.53
Clorgyline	18.74 ± 0.096	–	–	− 5.773	–
Pargyline	–	20.04 ± 0.095	–	–	− 6.061

^a^Values related for the evaluated compound absorption which provide 50% inhibition of MAO-A and MAO-B, action, and are the mean SEM; statistical significance: p < 0.05 against the equivalent IC_50_ values achieved against MAO-A and MAO-B, as identified through ANOVA/Dunnett’s test

^b^Selectivity index = IC_50_ (MAO-A)/IC_50_ (MAO-B)

**Fig. 3:**
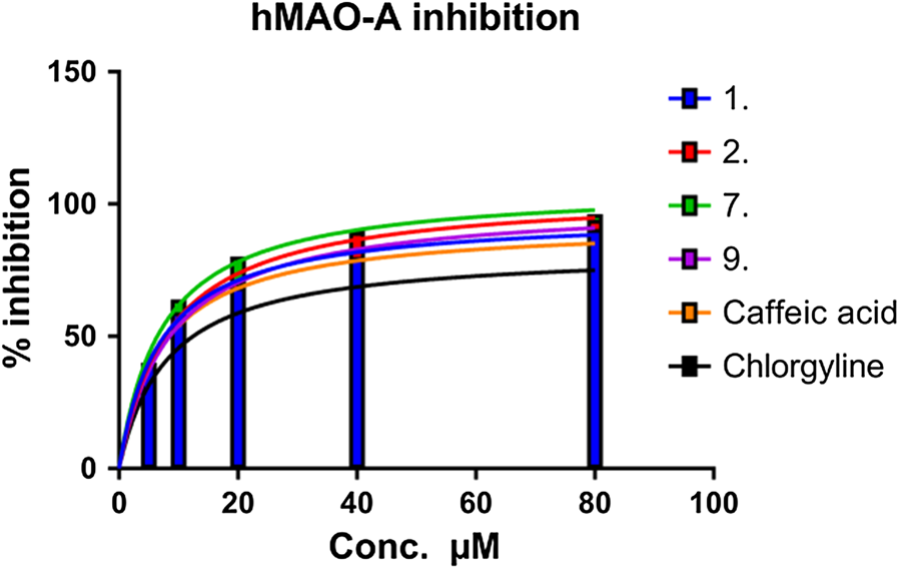
Concentration-dependent hMAO-A inhibition by most active compounds

The most active compound **7** was found as the hMAO-A inhibitor with IC_50_ values of 07.03 ± 0.022 µM with good selectivity (SI = 0.291) towards MAO-A. Due to the presence of eugenol as esterifies with caffeic acid at the 3rd position, it enhanced the hMAO-A potential. Another compound **9** also shown considerable inhibition for hMAO-A estimated IC_50_ value of 10.33 ± 0.012 µM and selectivity ratio of 0.379 for hMAO-A. It was suggested that substitution with halogen at the anilide ring, especially at the 2nd and 4th position strengthen the hMAO-selectivity for e.g. compounds **2**, **1**, **3** while at 3rd position (compound **4**) it does not show remarkable potential. Among the natural substituted esters derivatives only compound **7** was found as a promising hMAO-A inhibitor, and compound **6** was found selective for hMAO-B. The possible reason for this could be the different structural features of the natural phenols. However, caffeic acid (SI = 0.512) and reference compound (Chlorgyline) exhibited IC_50_ value for hMAO-A of 11.72 ± 0.044 µM and 18.74 ± 0.096 µM respectively (Fig. 3).

In case of hMAO-B, only two compounds **5** and **10** appeared as potential hMAO-B inhibitors with the IC_50_ value of 10.80 ± 0.024 µM and 12.95 ± 0.046 µM with good selectivity (SI = 4.653 and 1.947) towards MAO-B respectively. Among all derivatives compound **11** has been the weak MAO-B inhibitor. The possible reason for hMAO-B inhibition could be the presence of naphthyl ring in compound **5** and **11** which may be responsible for better interaction within the substrate cavity of hMAO-B. The caffeic acid and reference compound (Pargyline) had shown hMAO-B inhibition with experimental IC_50_ values of 22.88 ± 0.009 µM and 20.04 ± 0.095 µM respectively. In general, the entire series of caffeic acid derivatives had shown the hMAO-A inhibition except compound **5**, **10**, and **11**.

### Enzyme kinetics of MAO-A and MAO-B

**Fig. 4:**
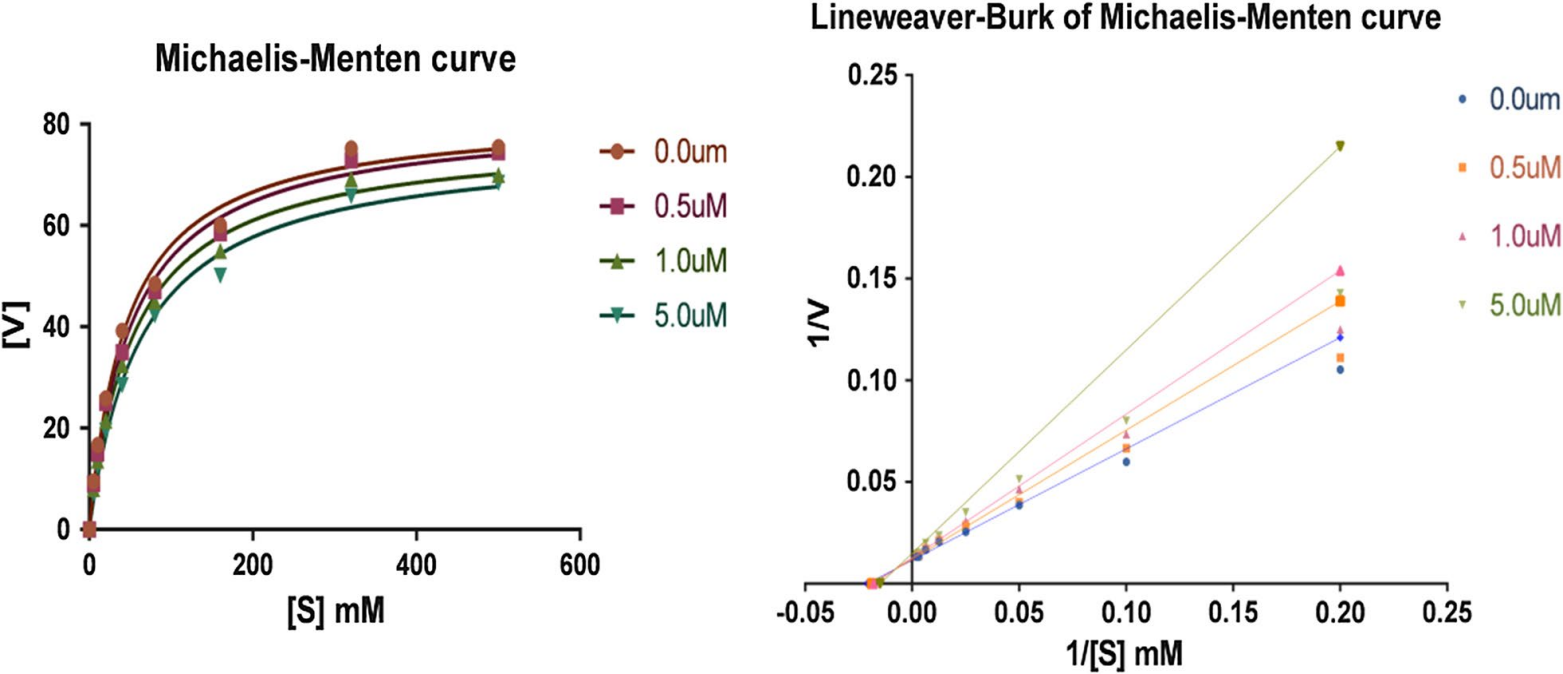
Lineweaver–Burk plot for oxidation of tyramine by MAO-A in the presence and absence of compound **7**

**Fig. 5:**
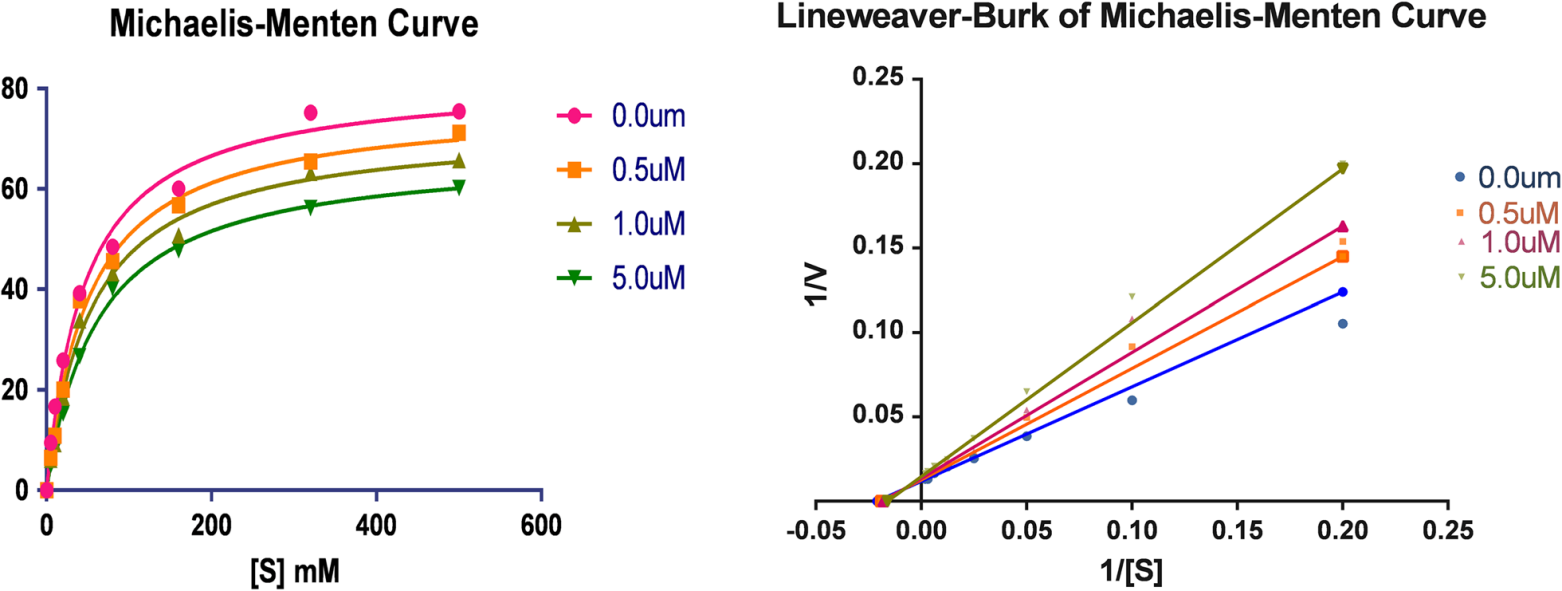
Lineweaver–Burk plot for oxidation of tyramine by MAO-B in the presence and absence of compound **5**

A set of Lineweaver–Burk plots was constructed in the presence and absence of different concentrations of compound **7** and **5** for hMAO-A and hMAO-B respectively. The graph comprises of five plots, every plot raised by quantifying MAO-B and MAO-A catalytic rate at various concentrations of substrate (0.5–5.0 µM). The resultant lines were found as linear and intersect on the y-axis indicated that compound 7 interacts within the active site of hMAO-A, via competitive inhibition mode (Fig. 4). In the case of compound **5**, the Lineweaver–Burk reciprocal plot obtained shows that compound **5** had the same *V*max value at several concentrations, but the *K*m value reduced with increasing concentration. Therefore, the inhibition of compound **9** against hMAO-A was indicated to be competitive, as shown in Fig. 5.

### DPPH radical scavenging activity

**Fig. 6:**
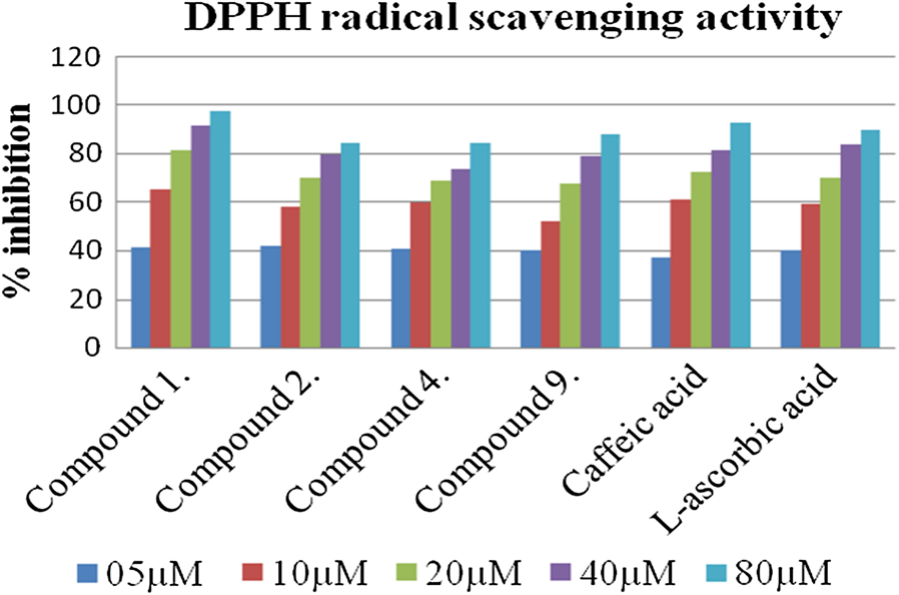
DPPH radical scavenging activity of most active compounds with respect to reference l-ascorbic acid

All the 12 synthesized compounds were subject for their evaluation of antioxidant profile using four models DPPH radical scavenging assay method (Table 4). In this screening, the compounds **1** and **2** were observed as most potent antioxidant candidates than that of reference (l-ascorbic acid) showing IC_50_ values as 06.39 ± 0.007 µM, 08.09 ± 0.042 µM, and 8.5.18 ± 0.009 µM (l-ascorbic acid) respectively. However compounds **4**, **9** were appeared as considerable antioxidants than other compounds of the series with IC_50_ values as 09.19 ± 0.001 µM, 10.57 ± 0.004 µM respectively. Electron withdrawing groups increases the efficiency to hydrogen release from the amino and phenolic group of caffeic acid derivatives. In case of compound **9** bearing extra phenolic ring than that of simple caffeic acid, could be the reason for improved scavenging action. Least activity was observed with the naphthyl substitutions to the caffeic acid compounds **5** (20.80 ± 0.003 µM) and **12** (15.37 ± 0.054 µM), because of the resonance thereby stabilize the molecules against the release of hydrogen, so these substitutions are not recommended further for the antioxidant molecules. However, caffeic acid and reference (l-ascorbic acid) exhibited IC_50_ values as 9.679 ± 0.013 µM and 8.5.18 ± 0.009 µM respectively (Fig. 6).

**Table 4:** DPPH radical scavenging of caffeic acid derivatives

Sr. no	IC_50_ (µM)^a^	Sr. no	IC_50_ (µM)^a^
1.	06.39 ± 0.007	8.	11.94 ± 0.025
2.	08.09 ± 0.042	9.	10.57 ± 0.004
3.	13.56 ± 0.003	10.	11.34 ± 0.078
4.	09.19 ± 0.001	11.	12.95 ± 0.031
5.	20.80 ± 0.003	12.	15.37 ± 0.054
6.	11.34 ± 0.001	Caffeic acid	9.679 ± 0.013
7.	11.22 ± 0.012	l-Ascorbic acid	8.5.18 ± 0.009

^a^Value are expressed as mean ± SEM, n = 3

### H_2_O_2_ radical scavenging activity

**Fig. 7:**
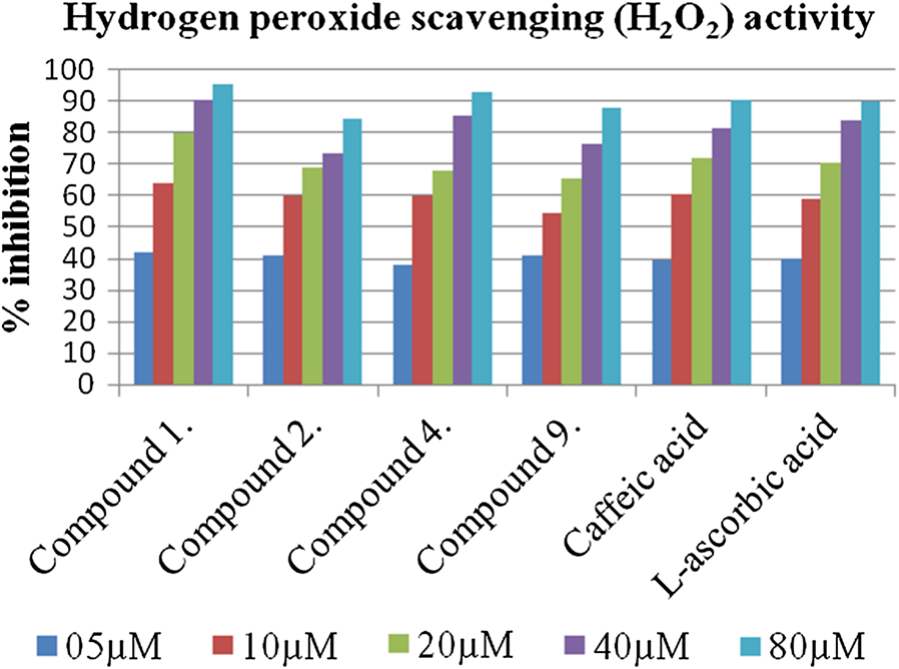
Hydrogen peroxide scavenging (H_2_O_2_) activity of most active compounds with respect to reference l-ascorbic acid

The behavior of titled compounds to scavenge H_2_O_2_ was measured to further validate the antioxidant potential (Table 5). Surprisingly, most active DPPH scavenger compound **1** was observed as potent antioxidant compound with IC_50_ values as 6.292 ± 0.007 µM while reference (l-ascorbic acid) exhibited IC_50_ values as 8.121 ± 0.082 µM. Compounds **2**, **4**, **9** have appeared as notable antioxidants than other compounds of the series with IC_50_ values as 8.544 ± 0.015 µM, 9.486 ± 0.017 µM, 9.907 ± 0.014 µM respectively. By comparing the tested amides and ester compounds, results showed amides (with electron withdrawing groups) were the most active one as the antioxidant. In the mechanism of H_2_O_2_ scavenging, the bond dissociation energies of the N–H and O–H bonds is an important parameter in evaluating the antioxidant action, because the weaker the N–H and O–H bonds the easier will be the reaction of free radical inactivation. The caffeic acid and reference (l-ascorbic acid) exhibited IC_50_ values as 8.868 ± 0.011 µM and 8.121 ± 0.082 µM respectively (Fig. 7).

**Table 5:** H_2_O_2_ radical scavenging activity of caffeic acid derivatives

Sr. no	IC_50_ µM^a^	Sr. no	IC_50_ µM^a^
1.	6.292 ± 0.007	8.	11.88 ± 0.008
2.	8.544 ± 0.015	9.	9.907 ± 0.014
3.	12.56 ± 0.021	10.	11.39 ± 0.042
4.	9.486 ± 0.017	11.	11.34 ± 0.045
5.	17.64 ± 0.005	12.	14.65 ± 0.026
6.	21.69 ± 0.034	Caffeic acid	8.868 ± 0.011
7.	12.78 ± 0.024	l-Ascorbic acid	8.121 ± 0.082

^a^Value are expressed as mean ± SEM, n = 3

### SAR (structure–activity relationship) studies

**Fig. 8:**
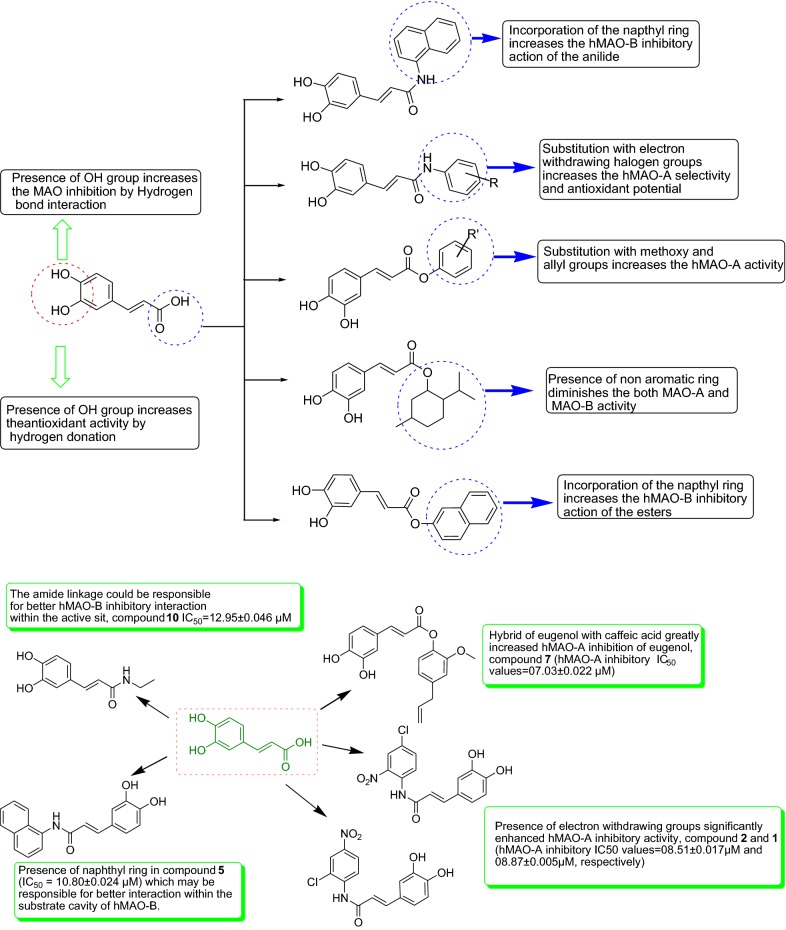
Careful insight into an examination of the MAO screening and antioxidant assay explicated of some SARs, along with few active compounds as significant MAO-A and MAO-B inhibitors

The structure–activity relationship of the synthesized caffeic acid derivatives with their anti-MAO and antioxidant activity results is summarized in Fig. 8.

1. The substitution of caffeic acid with the aromatic ring having electron withdrawing groups (compounds **2**, **1**, **3**, **4**) increased the hMAO-A affinity and selectivity.
2. Presence of hydroxyl group (**1**–**12**) improved the antioxidant activity.
3. Presence of non-aromatic ring to the caffeic acid moiety did not improved anti-MAO as well as antioxidant activity.
4. Substitution with electron withdrawing group such as *chloro*, *nitro* on anilide ring portion of compound **2**, **1** increased the antioxidant potential.
5. Incorporation of naphthyl ring (compound **5** and **11**) to caffeic acid increased hMAO-B inhibition significantly.

### Molecular docking studies of MAO inhibitors

**Fig. 9:**
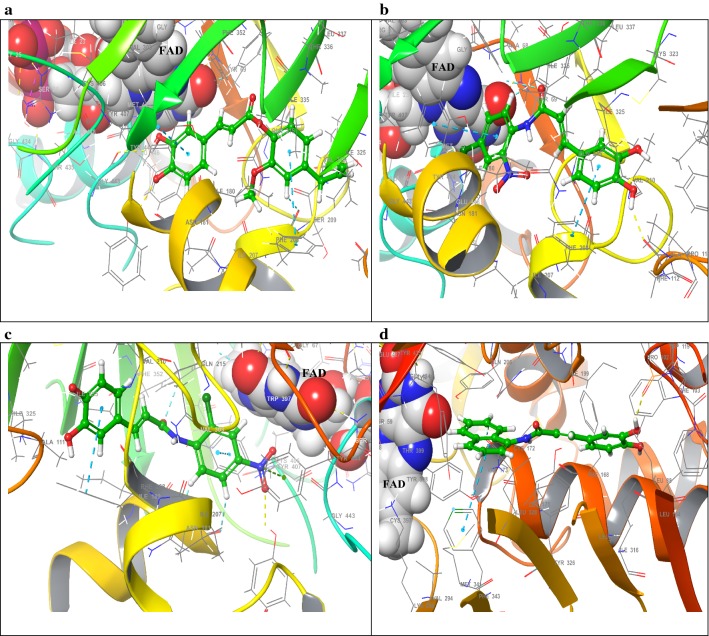
Graphical illustration of predicted binding mode of compounds 7, 2, 1 (within the hMAO-A binding site) and 5 (within the hMAO-B binding site) as into **a**–**d** respectively. Ligands are depicted in green carbon sticks. Major interacting amino acid residues are as gray carbon sticks, while FAD is in space-fill. Blue dotted lines specify ligand–enzyme π–π stacking interaction; yellow dotted lines signify ligand–enzyme intermolecular hydrogen bonds; green dotted lines signify ligand–enzyme π–cation stacking interaction. Hybrid caffeic acid derivatives as monoamine oxidases inhibitors: synthesis, radical scavenging activity, molecular docking studies and in silico ADMET analysis

With the aim to insight the binding orientation of the MAO inhibitory compounds, molecular docking technique was carried out by Mastero 11.5. In view of the inhibitory behavior and docking experiments of the compound **7** collectively; it can be assumed that (E)-4-allyl-2-methoxyphenyl 3-(3,4-dihydroxyphenyl) acrylate structure is an appropriate scaffold for the MAO-A enzyme; bulkier substituents than methyl such as 4-allyl on the methoxyphenyl moiety made contribution towards selectivity; and the presence of a hydrogen bond acceptor methoxy group at the aromatic ring increases the selectivity and potency towards MAO-A. The docking pose of compound **7** showed astonishing molecular interactions among ligand and protein complex by forming two hydrogen bonds between Tyr197 and Gly443 with phenolic OH of the ligand via inter-plane distance of ~ 2.45 Å and ~ 3.21 Å respectively (Fig. 9). The formation of two π–π stacking bonds between aromatic cage formed by Tyr407 and Tyr444 residues contributed firm hold of the molecule within the compact substrate cavity. Another edge to face π–π stacking was established between the 4-allyl-2-methoxyphenyl unit and Phe208 within MAO-A via inter-plane distance of ~ 5.09 Å, supported the ligand from the peripheral site of the substrate cavity. Moreover, the hydrophobic residues such as Ile335, Cys323, Leu97, Ile329, Phe108, Ala111, and Ile180 surrounded the aromatic acrylate chain and 4-allyl-2-methoxyphenyl unit to near to the N 5th side of the FAD. Polar residues such as Gln215, Thr336 were found engage towards ester linkage of compound **7**.

The binding orientation of compound **2** within the catalytic site of MAO-A exhibited a backbone hydrogen bonding with Ala111 through phenolic OH group with an inter-plane distance of ~ 1.87 Å. The anilide ring bearing (2-NO_2_, and 4-Cl) formed two π–π stacking infractions with an aromatic case of Phe353, and Tyr407 within the active site of hMAO-A through inter-plane distance about ~ 5.48 Å and ~ 4.30 Å respectively. However, another π–π stacking infraction has appeared between the phenolic ring of caffeic acid via Phe208 with inter-plane distance as ~ 1.89 Å. Additionally, the inhibitors were stabilized by hydrophobic residues of the substrate binding domain via Leu97, Val210, Phe112, Phe108, Ile325, Tyr444, Ile180 amino acid residues (Fig. 9).

Assessment of the best docking pose of compound **1** with hMAO-A protein revealed the formation of one edge to face π–π stacking between Phe208 and the anilide ring bearing (2-Cl, and 4-NO_2_). The OH group of terminal phenyl ring established backbone hydrogen bond with Tyr197 at an inter-plane distance of ~ 1.89 Å. Another π–π stacking interaction emerged between Tyr444 and the phenolic ring of caffeic acid near the vicinity of the FAD cofactor (Fig. 9). Interestingly, a π–cation interaction with Phe208 and the 2-NO_2_ group of the anilide ring of compound **1** at an interplane distance of ~ 4.67 Å. Additionally, the molecule is also stabilized by hydrophobic residues such as Tyr69, Met350, Phe352, Ile335, Ile180, Ala111, Val210, Cys323. Polar residues Thr336 and Gln215 were held as the supporter to anilide bond of the compound **1**.

In general, all of the evaluated inhibitors occupied the dynamic core of the MAO-A enzyme and were encircled by residues Tyr 444, Cys 323, Tyr 69, Tyr 407, Phe 352, Ile 335, Asn 181, Ile 180, Ile 325, Val210, Phe 208, Gln 215, and FAD. For the majority of the evaluated inhibitors, the phenolic moiety engaged within the compact cavity expanded toward flavin cofactor, while the ester and anilide units were situated near the opening of the substrate site. All of the tested compounds revealed one or more interactions as hydrogen bonding except compound **12**.

The explored the binding modes and interactions of compound **5** within the active site of the hMAO-B, shown one backbone hydrogen bond (at an inter-plane distance of ~ 1.90 Å) with Pro102 of hMAO-B and OH group of phenolic ring of caffeic acid near the “gate keeper” residue Ile199. A notable edge to face π–π stacking interaction emerged between Phe343 with a naphthyl ring of anilide unit of compound **5** with an inter-plane distance of ~ 5.21 Å (Fig. 9). The binding orientation of all compound **5** within MAO-B navigated both the cavities (entrance and substrate cavity), surrounded by residues Ile199, Ile198, Phe168, Tyr326, and Leu167 at entrance, and in substrate cavity, surrounded by Tyr435, Gln206, Cys172, Phe343, Tyr398, and Tyr60 residues, comparable to the binding mode of co-crystallized safinamide (MAO-B inhibitor).

Surprisingly, all compounds showed interactions as hydrogen bonding except compounds **8**, and 12 consisting carvacrol and menthol as substituted esters. Another weak hMAO-B inhibitor compound **10** showed a hydrogen bonding with Pro102 with phenolic OH with an inter-plane distance of ~ 2.03 Å. A polar residue Gln206 was found embedded towards the amide linkage of the molecule. In particular, for most of the docked compounds, the “gate keeper” residue Ile199 and Phe168 allowed only phenolic ring into the substrate site for hydrogen bonding with Pro102 except compounds **8** and **12**. All most all of the tested compounds appeared to be surrounded by closed residues such as Leu88, Phe99, Phe103, Trp119, Leu164, Phe168, Pro104, Leu171, Phe103 in the bipartite substrate cavity of the hMAO-B, whereas the residues of entrance cavity Tyr 435, Tyr 398, Tyr 188, Cys172, Tyr 326, Phe343, Met341, Leu328 enclosed the aromatic acrylate chain of the caffeic acid derivatives.

### In silico ADME profiling

An assessment of the information specified in Table 6 depicts that all synthesized compounds comply with Rule of five [25] and their molecular descriptors such as LogP, hydrogen bond acceptor (HBA), and hydrogen bond donor (HBD), topological polar surface area (TPSA), molecular weight (MW), lie in the satisfactory range for drug-like characteristics. Total surface pertains to the number of polar atoms, and this descriptor is reported to associates with passive molecular transport via membranes and, consequently, permits the calculation of transport characteristics of drugs within intestines as well as blood–brain barrier passage. In addition, according to Veber et al. [26], for exhibiting excellent bioavailability is presumptive for analogs with TPSA of ≤ 140 Å and rotatable bonds ≤ 10. Presence of maximum rotatable bonds in ligand imparts flexibility and makes ligands more adjustable for competent interaction with the active site cavity. In the present study, compounds **(1**–**12)** exhibited an acceptable drug-like profile, which is a sign of better bioavailability by the oral route.

**Table 6:** All descriptors are generated by the Quickprop software of Schrödinger

Sr. no	Mol. wt	TPSA	No. of rotatable bonds	DonorHB	AccptHB	QPlogPo/w	QPlogBB	QPPMDCK	QPPCaco
1.	334.71	115.38	4	3	5	1.773	− 2.173	36.004	38.398
2.	334.71	115.38	4	3	5	2.391	1.258	263.91	226.62
3.	334.17	69.55	3	3	4	3.31	1.363	32.746	78.746
4.	273.26	69.55	3	3	4	2.061	1.307	176.66	226.74
5.	305.33	69.55	3	3	4	2.786	1.476	120.18	270.07
6.	314.29	93.07	6	2	6	1.498	2.105	35.928	88.37
7.	326.35	76.00	7	2	4	3.421	1.451	222.06	476.59
8.	312.37	66.76	5	2	4	3.578	1.216	258.22	547.98
9.	272.26	86.99	4	3	4	1.546	1.759	57.484	136.50
10.	207.23	69.55	3	3	4	1.086	1.233	147.94	327.31
11.	306.32	66.76	4	2	4	3.217	1.222	208.85	450.32
12.	318.41	66.76	5	2	3	3.783	1.237	225.48	483.39

## Experimental

### Materials and methods

Unless otherwise noted, the chemicals required for synthesis and antioxidant activity were purchased from Hi-media Laboratories. The biological hMAO activity evaluation of the test drugs was examined by quantifying their action on the generation of H_2_O_2_ by p-tyramine (general substrate for hMAO-B and hMAO-A), utilizing the Amplex Red MAO assay kit (Sigma USA) and MAO isoforms (microsomal) obtained from insect cells (BTI-TN-5B14) expressed as recombinant baculovirus consisting cDNA probes for hMAO-A or hMAO-B. Reactions were monitored by thin layer chromatography TLC executed over silica gel precoated plates (0.25 mm) purchased from Merck, envisioned of single spots was carried in iodine and UV chambers, in mobile media TLC- Benzene:Chloroform (7:3). Melting points were recorded on Sonar melting point apparatus in open capillary tubes. The nuclear magnetic resonance (NMR) spectra ^1^H NMR and ^13^C NMR spectra were confirmed in DMSO and deuterated CDCl_3_ respectively on Bruker Avance II 400 NMR spectrometer at a frequency of 400 MHz downfield to tetramethylsilane standard. Coupling constants (J) were reported in Hertz (Hz) and chemical shifts were depicted as d (parts per million). Infrared (IR) spectra were recorded on Perkin Elmer FTIR spectrophotometer by using KBr pellets technique. Waters Micromass Q-ToF Micro instrument was used for Mass spectra recording.

### General procedure for the synthesis of caffeic acid chloride

In a round bottom flask (250 mL) thionyl chloride (10 mL) was added to caffeic acid (20 mmol, 2.46 g) in the presence of diethyl ether as a solvent and few drops of pyridine as the catalyst. The above reaction mixture was refluxed with stirring at 80 °C for 1–4 h. The progress of the reaction was scrutinized by TLC. The surplus amount of thionyl chloride was removed under low-pressure distillation. Lastly, the purity of the product was detected by single spot TLC under UV lamp and IR: peak shifted 1640 (carboxylic) to 1768 (acid chloride); Yield: 78%; MP: 155–157 °C.

### General procedure for the synthesis of natural hybrid caffeic acid esters

Natural hybrid esters derived from caffeic acid were prepared by refluxing aromatic alcohols with a solution of caffeic acid chloride (0.05 mol) with ether (50 mL) at 70–80 °C for 8–10 h (Scheme 1). The mixture was refluxed on the water bath until the evolution of hydrogen chloride gas was stopped consequently the reaction completion was confirmed by single spot TLC. Finally, this mixture was placed at room temperature for solvent evaporation which yielded the crude product. This was extracted with ether (50 mL) to obtain ester, which was subjected to final recrystallization by acetone.

### General procedure for the synthesis of amides

The solutions of equivalent amine/aniline (0.1 mol) in ether (50 mL) dropped to a caffeic acid chloride solution (0.1 mol) in ether (50 mL) kept at 0–10 °C temperature (Scheme 1). This reaction mixture was stirred up to 40 min and yielded precipitates were separated by filtration. Amides formed as crude precipitates were recrystallized with ethanol and further acidified with 5% hydrochloric acid and then treated with 4% sodium carbonate to remove water and residual aniline finally the extracted anilides were recrystallized with methanol.

### Spectral data

#### (E)-*N*-(2-chloro-4-nitrophenyl)-3-(3,4-dihydroxyphenyl)acrylamide **1**)

*R*_*f*_ TLC mobile phase: Benzene:Chloroform (7:3) = 0.67; (%) Yield = 67.3; M.P = 230–231 °C; IR (KBR pellets) cm^−1^; 3475 (O–H str., Ar), 3354 (N–H str., amide), 1627 (C=O str., amide), 1504 (NO_2_ str., nitro), 648 (Cl str., chloro); ^1^H NMR (400 MHz, DMSO-*d*_6_) δ = 8.27 (d, *J* = 1.5 Hz, 2H), 8.06 (dd, *J* = 7.5, 1.5 Hz, 2H), 7.90 (d, *J* = 7.5 Hz, 2H), 7.43 (dd, *J* = 14.9, 0.8 Hz, 2H), 7.06 (d, *J* = 1.5 Hz, 2H), 7.02 (s, 1H), 6.99–6.93 (m, 3H), 6.77 (d, *J* = 7.5 Hz, 2H); ^13^C NMR (400 MHz, CDCl_3_) δ = 168.5, 152.5, 145.9, 142.8, 139.8, 136.3, 128.2, 123.8, 121.6, 120.5, 119.8, 118.8, 117.3, 115.3, 112.8; MS ES + (ToF): m/z 333.34 [M^+^+2]; CHN: Calc. C_15_H_11_ClN_2_O_5_: C, 53.83; H, 3.31; N, 8.37; Found: C, 53.79; H, 3.27; N, 8.20.

#### (E)-*N*-(4-chloro-2-nitrophenyl)-3-(3,4-dihydroxyphenyl)acrylamide **2**)

*R*_*f*_ TLC mobile phase: Benzene:Chloroform (7:3) = 0.73; (%) Yield = 73.2; M.P = 200–202 °C; IR (KBR pellets) cm^−1^; 3437 (O–H str., Ar), 3313 (N–H str., amide), 1629 (C=O str., amide), 1536 (NO_2_ str., nitro), 721 (Cl str., chloro); ^1^H NMR (400 MHz, DMSO-*d*_6_) δ = 8.15 (d, *J* = 1.5 Hz, 2H), 7.86 (d, *J* = 7.5 Hz, 2H), 7.61 (dd, *J* = 7.5, 1.5 Hz, 2H), 7.41 (dd, *J* = 14.9, 0.8 Hz, 2H), 7.26 (d, *J* = 1.4 Hz, 2H), 7.12 (s, 1H), 6.98–6.95 (m, 3H), 5.03 (d, *J* = 7.5 Hz, 2H); ^13^C NMR (400 MHz, CDCl_3_) δ = 167.9, 150.5, 149.9, 138.8, 137.7, 135.5, 132.5, 130.1, 127.2, 124.4, 123.3, 120.8, 118.3, 115.3, 114.8; MS ES + (ToF): m/z 333.09 [M^+^+2]; CHN: Calc. C_15_H_11_ClN_2_O_5_: C, 53.83; H, 3.31; N, 8.37; Found: C, 53.80; H, 3.27; N, 8.33.

#### (E)-*N*-(4-bromophenyl)-3-(3,4-dihydroxyphenyl)acrylamide **3**)

*R*_*f*_ TLC mobile phase: Benzene:Chloroform (7:3) = 0.8; (%) Yield = 59.9; M.P = 167–167.8 °C; IR (KBR pellets) cm^−1^; 3536 (O–H str., Ar), 3174 (N–H str., amide), 1637 (C=O str., amide), 572 (Br str., bromo); ^1^H NMR (400 MHz, DMSO-*d*_6_) δ = 8.61–8.54 (m, 8H), 7.45–7.37 (m, 2H), 7.09 (d, *J* = 1.4 Hz, 2H), 7.07 (s, 1H), 7.02–6.94 (m, 3H), 5.14 (d, *J* = 7.5 Hz, 2H); ^13^C NMR (400 MHz, CDCl_3_) δ = 168.7, 151.5, 144.9, 143.8, 140.4, 135.8, 127.2, 121.7, 120.8, 118.5, 115.8, 111.3, 110.8; MS ES + (ToF): m/z 333.00 [M^+^+2]; CHN: Calc. C_15_H_12_BrNO_3_: C, 53.91; H, 3.62; N, 4.19; Found: C, 53.89; H, 3.60; N, 4.16.

#### (E)-3-(3,4-dihydroxyphenyl)-*N*-(3-fluorophenyl)acrylamide **4**)

*R*_*f*_ TLC mobile phase: Benzene:Chloroform (7:3) = 0.57; (%) Yield = 71.6; M.P = 189–186 °C; IR (KBR pellets) cm^−1^; 3374 (O–H str., Ar), 3044 (N–H str., amide), 1632 (C=O str., amide), 1192 (F str., fluro); ^1^H NMR (400 MHz, DMSO-*d*_6_) δ = 7.60 (dt, *J* = 7.5, 1.5 Hz, 2H), 7.13–7.03 (m, 6H), 6.95 (d, *J* = 1.4 Hz, 2H), 6.92 (s, 1H), 6.91–6.87 (m, 3H), 6.77 (ddt, *J* = 8.0, 7.5, 1.5 Hz, 2H), 6.13 (d, *J* = 7.5 Hz, 2H); ^13^C NMR (400 MHz, CDCl_3_) δ = 165.1, 163.8, 161.3, 150.5, 147.9, 144.8, 138.3, 131.3, 127.2, 124.8, 120.5, 119.5, 116.3, 114.8, 112.1, 110.9, 108.22, 108.2; MS ES + (ToF): m/z 272.23 [M^+^+2]; CHN: Calc. C_15_H_12_FNO_3_: C, 74.74; H, 4.95; N, 4.59; Found: C, 74.71; H, 4.92; N, 4.55.

#### (E)-3-(3,4-dihydroxyphenyl)-*N*-(naphthalen-1-yl)acrylamide **5**)

*R*_*f*_ TLC mobile phase: Benzene:Chloroform (7:3) = 0.71; (%) Yield = 82.1; M.P = 176–176.3 °C; IR (KBR pellets) cm^−1^; 3498 (O–H str., Ar), 3258 (N–H str., amide), 1627 (C=O str., amide), 1595 (C=C skeletal str., naphthyl); ^1^H NMR (400 MHz, DMSO-*d*_6_) δ = 7.88–7.81 (m, 2H), 7.72–7.65 (m, 2H), 7.64–7.57 (m, 2H), 7.54–7.51 (m, 3H), 7.52–7.42 (m, 3H), 7.39 (d, *J* = 0.7 Hz, 1H), 7.20 (td, *J* = 7.5, 0.5 Hz, 2H), 7.10 (d, *J* = 1.5 Hz, 2H), 7.01 (s, 1H), 7.00–6.93 (m, 3H), 6.78 (d, *J* = 7.5 Hz, 2H); ^13^C NMR = (400 MHz, CDCl_3_) δ 167.1, 149.5, 147.9, 141.8, 136.5, 135.1, 127.8, 126.4–126.1 (m), 125.6, 125.4, 124.2, 123.8, 119.3, 117.2, 114.8; MS ES + (ToF): m/z 272.23 [M^+^+1]; CHN: Calc. C_19_H_15_NO_3_: C, 65.93; H, 4.43; N, 5.13; Found: C, 65.89; H, 4.41; N, 5.11.

#### (E)-4-formyl-2-methoxyphenyl 3-(3,4-dihydroxyphenyl)acrylate **6**)

*R*_*f*_ TLC mobile phase: Benzene:Chloroform (7:3) = 0.49; (%) Yield = 75.5; M.P = 150–151 °C; IR (KBR pellets) cm^−1^; 3432 (O–H str., Ar), 1725 (C=O str., ester), 1644 (C=C skeletal str., alkene), 2839, 2734 (C–H str., aldehydes), 1234 (C–O str., ester), 1150 (C–O–C assym. str., Ar–O–CH_3_); ^1^H NMR (400 MHz, DMSO-*d*_6_) δ = 9.83 (t, *J* = 0.5 Hz, 1H), 7.69 (dd, *J* = 15.0, 0.8 Hz, 1H), 7.49–7.45 (m, 2H), 7.36 (d, *J* = 7.4 Hz, 1H), 7.06 (d, *J* = 1.6 Hz, 1H), 6.96 (ddd, *J* = 7.5, 1.5, 0.6 Hz, 1H), 6.89 (d, *J* = 15.1 Hz, 1H), 6.78 (d, *J* = 7.5 Hz, 1H), 3.87 (s, 3H); ^13^C NMR (400 MHz, CDCl_3_) δ = 194.3, 163.1, 152.6, 149.5, 148.2, 146.5, 141.6, 135.6, 127.8, 123.6, 122.6, 120.5, 117.3, 115.4, 115.8, 110.1 57.1; MS ES + (ToF): m/z 313.05 [M^+^+1]; CHN: Calc. C_17_H_14_O_6_: C, 64.97; H, 4.49; Found: C, 64.95; H, 4.46.

#### (E)-4-allyl-2-methoxyphenyl 3-(3,4-dihydroxyphenyl)acrylate **7**)

*R*_*f*_ TLC mobile phase: Benzene:Chloroform (7:3) = 0.67; (%) Yield = 73.6; M.P = 161–161.7 °C; IR (KBR pellets) cm^−1^; 3436 (O–H str., Ar), 1732 (C=O str., ester), 1268 (C–O str., ester), 1124 (C–O–C assym. str., Ar–O–CH_3_); ^1^H NMR (400 MHz, DMSO-*d*_6_) δ 8.16 (dd, *J* = 14.9, 0.8 Hz, 2H), 7.10–7.05 (m, 4H), 6.91 (ddd, *J* = 7.5, 1.5, 0.6 Hz, 2H), 6.84 (d, *J* = 15.1 Hz, 2H), 6.79–6.75 (m, 6H), 5.94 (ddt, *J* = 16.8, 10.1, 6.2 Hz, 2H), 5.19–5.11 (m, 3H), 5.08 (dt, *J* = 2.1, 1.0 Hz, 1H), 3.84 (s, 6H), 3.32 (dp, *J* = 6.2, 1.0 Hz, 4H); ^13^C NMR (400 MHz, CDCl_3_) δ 165.1, 153.3, 149.5, 145.2, 142.6, 140.2, 139.8, 138.2, 127.8, 123.6, 121.6, 119.8, 117.3, 117.3, 114.48, 114.8, 113.2, 55.9, 40.4; MS ES + (ToF): m/z 325.05 [M^+^+1]; CHN: Calc. C_19_H_18_O_5_: C, 69.93; H, 5.56; Found: C, 69.90; H, 5.54.

#### (E)-5-isopropyl-2-methylphenyl 3-(3,4-dihydroxyphenyl)acrylate **8**)

*R*_*f*_ TLC mobile phase: Benzene:Chloroform (7:3) = 0.79; (%) Yield = 69.5; M.P = 182–182.6 °C; IR (KBR pellets) cm^−1^; 3394 (O–H str., Ar), 1875 (C=O str., ester), 1616 (C=C skeletal str., alkene), 1255 (C–O str., ester); ^1^H NMR (400 MHz, DMSO-*d*_6_) δ = 8.52 (dd, *J* = 14.9, 0.8 Hz, 1H), 7.49 (d, *J* = 1.6 Hz, 1H), 7.01 (dq, *J* = 7.8, 0.9 Hz, 1H), 6.83–6.74 (m, 2H), 6.64–6.23 (m, 2H), 6.17 (d, *J* = 7.5 Hz, 1H), 2.92 (dtt, *J* = 13.5, 6.7, 0.8 Hz, 1H), 2.18 (d, *J* = 1.0 Hz, 3H), 1.28 (d, *J* = 6.8 Hz, 6H); ^13^C NMR (400 MHz, CDCl_3_) δ = 168.2, 150.4, 148.5, 145.2, 145.02, 144.8, 129.7, 128.4, 126.8, 122.7, 118.5, 116.3, 114.5, 112.0, 33.2, 23.9, 15.9; MS ES + (ToF): m/z 311.05 [M^+^+1]; CHN: Calc. C_19_H_20_O_4_: C, 73.06; H, 6.45; Found: C, 73.02; H, 6.41.

#### (E)-3-hydroxyphenyl 3-(3,4-dihydroxyphenyl)acrylate **9**)

*R*_*f*_ TLC mobile phase: Benzene:Chloroform (7:3) = 0.65; (%) Yield = 63.9; M.P = 198–198.4 °C; IR (KBR pellets) cm^−1^; 3375 (O–H str., Ar), 1810 (C=O str., ester), 1595 (C=C skeletal str., alkene), 1255 (C–O str., ester); ^1^H NMR (400 MHz, DMSO-*d*_6_) δ = 7.71–7.62 (m, 2H), 7.16 (tt, *J* = 7.6, 0.9 Hz, 2H), 7.06 (d, *J* = 1.5 Hz, 2H), 6.99–6.89 (m, 4H), 6.86 (dd, *J* = 1.5, 0.7 Hz, 3H), 6.86–6.77 (m, 4H), 6.77 (s, 1H), ^13^C NMR (400 MHz, CDCl_3_) δ = 169.9, 156.5, 152.3, 150.5, 146.2, 143.2, 129.9, 125.8, 123.6, 118.3, 118.1, 114.8, 112.3, 110.7, 107.1; MS ES + (ToF): m/z 271.02 [M^+^+1]; CHN: Calc. C_15_H_12_O_5_: C, 66.17; H, 4.44; Found: C, 66.14; H, 4.41.

#### (E)-3-(3,4-dihydroxyphenyl)-*N*-ethylacrylamide **10**)

*R*_*f*_ TLC mobile phase: Benzene:Chloroform (7:3) = 0.52; (%) Yield = 72.6; M.P = 194–194.2 °C; IR (KBR pellets) cm^−1^; 3464 (O–H str., Ar), 3278 (N–H str., amide), 1647 (C=O str., amine); ^1^H NMR (400 MHz, DMSO-*d*_6_) δ = 7.40–7.31 (m, 1H), 7.08–7.04 (m, 1H), 6.96 (ddd, *J* = 7.5, 1.5, 0.6 Hz, 1H), 6.78 (d, *J* = 7.5 Hz, 1H), 6.51 (d, *J* = 15.1 Hz, 1H), 3.24 (q, *J* = 8.0 Hz, 2H), 1.24 (t, *J* = 8.0 Hz, 3H); ^13^C NMR (100 MHz, Chloroform-*d*) δ = 167.9, 148.9, 148.5, 147.2, 145.2, 133.9, 131.5, 129.3, 127.5, 126.8, 126.4, 122.6, 121.2, 116.3, 116.1, 115.7, 115.8; MS ES + (ToF): m/z 206.03 [M^+^+1]; CHN: Calc. C_11_H_13_NO_3_: C, 63.76; H, 6.32; N, 6.76; Found: C, 63.74; H, 6.29; N, 6.73.

#### (E)-naphthalen-2-yl 3-(3,4-dihydroxyphenyl)acrylate **11**)

*R*_*f*_ TLC mobile phase: Benzene:Chloroform (7:3) = 0.8; (%) Yield = 75.4; M.P = 176–176.9 °C; IR (KBR pellets) cm^−1^; 3275 (O–H str., Ar), 1780 (C=O str., ester), 1601 (C=C skeletal str., naphthyl), 1216 (C–O str., ester); ^1^H NMR (400 MHz, DMSO-*d*_6_) δ = 7.88 (ddd, *J* = 7.5, 1.4, 0.6 Hz, 2H), 7.75 (dtd, *J* = 7.4, 1.4, 0.5 Hz, 2H), 7.71–7.62 (m, 4H), 7.58–7.48 (m, 4H), 7.45 (tdd, *J* = 7.4, 1.5, 0.5 Hz, 2H), 7.23 (ddd, *J* = 7.5, 1.5, 0.5 Hz, 2H), 7.07 (d, *J* = 1.5 Hz, 2H), 6.97 (ddd, *J* = 7.5, 1.5, 0.6 Hz, 2H), 6.86–6.77 (m, 3H), 6.77 (s, 1H); ^13^C NMR (400 MHz, CDCl_3_) δ = 166.3, 150.4, 150.5, 148.2, 144.2, 134.9, 130.5, 127.3, 126.5, 124.8, 124.4, 123.6, 120.2, 116.36, 115.1, 114.7, 114.8; MS ES + (ToF): m/z 305.05 [M^+^+1]; CHN: Calc. C_19_H_14_O_4_: C, 74.50; H, 4.61; Found: C, 74.47; H, 4.59.

#### (E)-2-isopropyl-5-methylcyclohexyl 3-(3,4-dihydroxyphenyl)acrylate **12**)

*R*_*f*_ TLC mobile phase: Benzene:Chloroform (7:3) = 0.74; (%) Yield = 78.3; M.P = 185–186 °C; IR (KBR pellets) cm^−1^; 3445 (O–H str., Ar), 1715 (C=O str., ester), 1612 (C=C skeletal str., Ar), 1198 (C–O str., ester), ^1^H NMR (400 MHz, DMSO-*d*_6_) δ = 7.47 (dd, *J* = 15.3, 0.8 Hz, 2H), 7.09 (d, *J* = 1.5 Hz, 2H), 6.95 (ddd, *J* = 7.5, 1.5, 0.6 Hz, 2H), 6.78 (d, *J* = 7.5 Hz, 2H), 6.15 (d, *J* = 15.1 Hz, 2H), 5.08 (q, *J* = 7.0 Hz, 2H), 2.04–1.87 (m, 4H), 1.87–1.82 (m, 4H), 1.80 (ddt, *J* = 8.5, 5.4, 1.5 Hz, 1H), 1.72–1.58 (m, 4H), 1.58–1.50 (m, 1H), 1.46–1.33 (m, 2H), 0.93–0.81 (m, 16H), ^13^C NMR (400 MHz, CDCl_3_) δ = 165.9, 149.5, 147.2, 146.9, 129.5, 122.7, 119.4, 117.1, 75.6, 46.4, 37.6, 34.1, 30.8, 27.8, 23.5, 21.6, 19.6; MS ES + (ToF): m/z 317.14 [M^+^+1]; CHN: Calc. C_19_H_26_O_4_: C, 71.67; H, 8.23; Found: C, 71.64; H, 8.19.

### Monoamine oxidase-A and MAO-B assays

All synthesized compounds were evaluated for their capability to inhibit the MAO-A and MAO-B isoforms of human MAO by a fluorometric method. The MAO inhibitory property of the test compounds was examined with the recombinant human enzymes using an Amplex Red Monoamine Oxidase Assay Kit (Sigma USA). The incessant peroxidase-linked photometric assay was performed on the fluorometer. Due to their limited aqueous solubility, a few compounds were solubilized in DMSO whose final concentration of 3.3% (v/v) does not affect MAO activity. Distilled water was used as a negative control. In brief, 0.1 mL of sodium phosphate buffer (0.05 M, pH 7.4) with the test new compounds/reference inhibitors in different concentrations and sufficient quantity of recombinant hMAO-A or hMAO-B requisite and in step to complete our investigational protocol with the equal reaction velocity, (hMAO-A: 1.1 mg protein; explicit activity: 150 nmol of p-tyramine oxidized to p-hydroxyphenylacetaldehyde/min/mg protein; hMAO-B: 7.5 mg protein; explicit activity: 22 nmol of p-tyramine changed/min/mg protein) were incubated for 15 min at 37 °C, placed in the dark fluorometric compartment. After incubation, the effect was attained through the addition of 200 mM Amplex Red reagent (final concentrations), 1 mM p-tyramine, and 1 U/mL horseradish peroxidase. Finally, the generation of hydrogen peroxide and, subsequently, of resorufin was measured at 37 °C in fluorescence reader (λ_emission_, 585 nm, λ_excitation_, 530 nm) after 15 min, by the time fluorescence amplified linearly. All the control experiments were performed concurrently by changing the reference inhibitors and test compounds with suitable dilutions in the solvents. Moreover, the activity of abovementioned evaluated compounds to alter the fluorescence produced in the combination reaction by (e.g. for reacting with Amplex Red reagent directly) non-enzymatic inhibition was identified by addition of these compounds to solutions having just the Amplex Red reagent in a sodium phosphate buffer. Clorgyline and pargyline were taken as the standard inhibitor at the same concentrations with the compounds. The fluorescence reading was computed by subtraction of the background activity and was evaluated from wells with all components not including the hMAO isoforms that was substituted by a sodium phosphate buffer solution. All data was processed in Microsoft Excel, to calculate IC_50_ values.

### Kinetic parameters of MAO activity

Kinetic constants of steady-state (*V*max, maximum rate, and *K*m, Michaelis constant,) were measured by studying the effects of substrate concentration on the primary rate of reaction of MAO-A or MAO-B in absence and presence of compounds at diverse concentrations. To examine the mode of action by synthesized derivatives all of them were solubilized in dimethyl sulfoxide, with the concentration of 1%, and utilized in a wide concentration range. Construction of Lineweaver–Burk plots illustrated the mode of MAO inhibition. Selectivity index SI (Ki (MAO-A)/Ki (MAO-B)) was also computed. Enzyme kinetics for the interaction of the compounds with the MAO enzymes was computed on the GraphPad Prism 7 software.

### Statistical analysis

Results in the studies are depicted as mean ± SEM (standard error mean). Experimental data were evaluated statistically by utilizing one-way analysis of variance (ANOVA). However, ANOVA revealed considerable difference as revealed by ANOVA/Dunnett’s test. *P *< 0.05 was regarded as to be statistically significant. The statistical evaluation was carried out by Graph Pad Prism 5.0 Version for Windows (San Diego, CA, USA).

## Molecular modeling studies

### Protein preparation

The 3D crystallographic structures of hMAO-A and hMAO-B were retrieved from the Protein Data Bank (PDB) exhibiting particular accession codes 2Z5X [27] and 2V5Z [28] for every protein structure. To the above proteins, the hydrogen was added and partial charges were assigned consequently, missing atoms were added to side chain and loops using the function “Protein Preparation Wizard” in Maestro 11.1 of the Schrodinger Suite. Finally, the protein was refined through energy minimization, utilizing OPLS-2005 as force field up to 0.30 Å root-mean-square deviation (RMSD) with respect to co-crystallized ligands to produce the docking grid box.

### Ligand preparation

The 3D structures were imported as mole file into the Maestro 11.1 of the Schrodinger Suite [29]. To prepare ligands structures were protonated and produced tautomeric structures at pH 7.4 by “LigPrep” module. Finally, the lowest energy conformations were attained by using the OPLS-2005 force field, prior to the molecular docking.

### GLIDE/ligand docking

Docking experiments were executed to rank and calculate the investigational and theoretical binding relationship. Output file generated after Grid were imported as an input for molecular docking against protein prepared targets in GLIDE. Flexible docking method was adopted in SP (Standard Precision) mode.

### Antioxidant activity

DPPH (2,2-diphenyl-1-picryl-hydrazyl-hydrate) free radical process is an antioxidant assay based on electron-transfer that turns violet color in ethanol. The antioxidant activity was based on free radical scavenging effect towards stable DPPH• radical upon spectrophotometric reaction according to the method reported in the literature [30]. Briefly, solutions of synthesized compounds were prepared in fifty milliliters of various concentrations (25, 50, 75, and 100) µg/mL dissolved in methanol, was added to 5 mL of a 0.004% methanol solution of DPPH. The stock solution was prepared with 0.01% DPPH• in ethanol/water (1:1). While sample solutions were prepared as 5 mL DPPH• solution was mixed with 5 mL ascorbic acid solution then shaken vigorously and kept in the dark at 37 °C. The samples were incubated at the 37 °C temperature for 30 min in the dark chamber subsequently, absorbance was measured. The marketable recognized antioxidant, ascorbic acid was utilized for contrast or as a positive control. Initially, the blank spectrum for ethanol/water was recorded subsequently the spectrophotometric titration was carried out with various concentrations of synthesized compounds. The absorbance was recorded at 517 nm with UV/Vis Epoch ELISA reader, and antioxidant activity was measured as a decrease in absorbance of DPPH•. The IC_50_ values of free radical DPPH, based on control reading were calculated by the following equation.

$$\% {\text{ inhibition of DPPH radical }} = \, \left[ {\left( {{\text{A}}_{\text{br}} - {\text{A}}_{\text{ar}} } \right)/{\text{A}}_{\text{br}} } \right] \, \times 100$$

where A_br_ is the absorbance prior to reaction and A_ar_ is the absorbance after reaction with DPPH•.

### Hydrogen peroxide scavenging (H_2_O_2_) assay

Amongst the reactive oxygen species (ROS), H_2_O_2_ is an essential radical as even though it is not lethal of its own accord, but can be changed to further even highly toxic radicals, for instance, OH^•^ by hypochlorous acid or Fenton reaction by the oxidases enzymes. A solution of H_2_O_2_ (40 µM) was added in phosphate buffer saline (50 µM pH 7.4). Different quantities of test and references compounds in DMSO were added to 2 mL of hydrogen peroxide solution in buffer solution. Subsequently, upon to 10 min, the absorbance was calculated at 230 nm against a blank solution including phosphate buffer without H_2_O_2_ [31]. The percentage of scavenging H_2_O_2_ was estimated by the formula in this way:

$$\% {\text{ Scavenging }}\left( {{\text{H}}_{ 2} {\text{O}}_{ 2} } \right) \, = \, \left[ {\left( {{\text{A}}_{\text{S}} - {\text{A}}_{\text{T}} } \right)/{\text{A}}_{\text{i}} } \right] \, \times 100$$

where A_S_ is the absorbance of control and A_T_ is the absorbance of the test.

## Conclusion

In conclusion, the above mentioned computational studies not only shed a light on the underlying mechanism of MAO-A and MAO-B inhibition but also afforded valuable insight for the rational development of inhibitory potency and specificity of modified caffeic acid derivatives to be discovered as the novel antidepressant and anti-Parkinsonian drug molecule. Moreover, the above mentioned most active compounds were found outstanding antioxidants towards DPPH and H_2_O_2_ with remarkable potential as compared to the reference compound.
